# Paracentral acute middle maculopathy in the immediate postoperative of cataract surgery

**DOI:** 10.5935/0004-2749.2021-0269

**Published:** 2023-03-20

**Authors:** Bruno Figueira Guimarães, Mariana Amaranto de Souza Damásio, Luciana Ferreira Brina, Gilberto Zulato Chaves Figueiredo, Jacques Ramos Houly

**Affiliations:** 1 Hospital de Olhos Hilton Rocha, Belo Horizonte, MG

**Keywords:** Tomography, optical coherence, Ophthalmologic surgical procedures, Postoperative complications, Risk factors, Cataract, Cataract extraction, Low vision, Eye health, Tomografia, coerência óptica, Procedimentos cirúrgicos oftalmológicos, Complicações pós-operatórias, Fatores de risco, Catarata, Extração de catarata, Baixa visão, Saúde ocular

## Abstract

This case report identified paracentral acute middle maculopathy as the cause of severe and irreversible vision loss after cataract surgery. Cataract surgeons should be aware of known risk factors for the development of paracentral acute middle maculopathy. In those patients, extra care regarding anesthesia, intraocular pressure, and some other aspects of cataract surgery must be taken. Paracentral acute middle maculopathy is currently understood as a clinical sign evident on spectral-domain optical coherence tomography, and it is probably evidence of deep ischemic insult to the retina. It should be a differential diagnosis in cases of marked low vision acuity associated with no fundus abnormalities in the immediate postoperative period, as demonstrated in the presented case.

## Abstract (pt)

O presente relato de caso identificou a maculopatia média aguda paracentral como a causa de baixa de acuidade visual severa e irreversível após cirurgia de catarata. Existem fatores de risco bem estabelecidos para o desenvolvimento da maculopatia média aguda paracentral que devem ser conhecidos pelos cirurgiões de catarata. Nesse contexto cirúrgico, precauções extras no tocante a procedimentos anestésicos, pressão intraocular e alguns outros aspectos da cirurgia devem ser consideradas. A maculopatia média aguda paracentral é descrita como um sinal clínico observado no exame de tomografia de coerência óptica por domínio espectral e se trata, provavelmente, da evidência de um evento isquêmico no tecido vascular retiniano. Esse diagnóstico deve ser cogitado nos casos de perda de acuidade visual súbita no pós-operatório imediato associada com exame fundoscópico normal, como evidenciado no caso apresentado.

## INTRODUCTION

Paracentral acute middle maculopathy (PAMM) was recognized, named, and characterized first in 2013^(1)^. It was referred as a hyperreflective parafoveal band at the level of the inner nuclear layer (INL) in the acute phase that progresses to thinning or atrophy of the retina’s layers. Patients usually present with sudden paracentral scotoma sometimes associated with severe vision loss.

Ischemic changes in the intermediate and deep capillary plexus of the retina are believed to play a major role in the pathophysiology of PAMM^(2,3)^. An extensive number of retinal and systemic vasculopathies, such as diabetic retinopathy^(4)^, central retinal vein occlusion^(5)^, retinal artery occlusion^(6,7,8,9)^, sickle cell anemia retinopathy^(10,11)^, and Purtscher retinopathy^(11,12,13)^ have been implied as possible etiologies of ischemic injury. Recently, it was reported as a postoperative adverse event in patients undergoing uncomplicated phacoemulsification with intraocular lens (IOL) implantation^(14)^.

## CASE REPORT

The patient was an 87-year-old woman who had systemic arterial hypertension, congestive heart failure, chronic kidney disease, previous history of acute myocardial infarction and myocardial revascularization procedures, hospital reports of blood transfusions in the previous years, and cataract in both eyes (OU).

In the preoperative evaluation for cataract surgery, biomicroscopy revealed anterior cortical (1+/4+) and nuclear (2+/4+) cataract OU. The best-corrected visual acuity (BCVA) values were 0.5 and 0.4 (Snellen) in the right eye (OD) and left eye (OS), respectively. The intraocular pressure (IOP) was 13 mmHg in OU. Fundoscopic examination showed applied retina bilaterally; increase in vessel tortuosity in OU; hard, small, and medium-sized drusen diffusely distributed in the posterior pole of OU; and hard and sparse small drusen in the macula of OS. The cup-to-disc ratio was 0.3, and no other nerve head alterations were observed in OU.

The patient underwent phacoemulsification with IOL in the OS and 35 days later in the OD. Both surgeries were performed by the same surgeon and with the same anesthetic procedure. The anesthetic block used in both eyes, by the same anesthesiologist who had extensive experience in ophthalmic blocks, was the peribulbar type. It contained 2 mL of 2% lidocaine hydrochloride with 1:200,000 epinephrine hemitartrate and 3 mL of 1% ropivacaine hydrochloride with hyaluronidase diluted at 1000 UTR in a 20-mL ampule of ropivacaine (50 units/mL). A total volume of 7 mL was infiltrated and distributed equally (3.5 mL) by superior and inferior temporal applications with a hypodermic needle (0.60 × 25 mm), with full insertion of the metallic part of the needle. After infiltration, a 20-g weight was used over the eye to aid in the dispersion of the medication. Regarding the anesthetic procedure, the patient was sedated with a bolus of 1 mL of a solution containing 3 mL of 15 mg/ 3 mL midazolam, 4 mL of 50 mcg/mL fentanyl citrate, and 3 mL of double-distilled water. Of this same solution, 1 mL was used in 250 mL of 0.9% saline with slow drip into the peripheral venous access.

The surgeries were performed by the same surgeon using the same surgical technique and phacoemulsification unit Infiniti®. No perioperative intercurrences were noted in both of them.

The procedure was performed as follows: antisepsis was performed with povidone-iodine (PVPI) 5% eye drops onto the eye and PVPI 10% onto the skin. No adrenaline or any other medication was used in the balanced salt solution infused into the patient’s eye during surgery. A 2.75-mm triplanar incision was made in the clear cornea, and no dye was used to stain the anterior capsule. A dispersive ophthalmic viscosurgical device Metilcelulose® was used, and capsulorrhexis was performed with utrata. The phaco chop surgical technique was employed. The IOL used was a three-piece TYPE7B model, and it was positioned inside the capsular bag using an injector. After the IOL implantation, the viscoelastic was removed with an irrigation-aspiration handpiece (IA). The surgery concluded with the hydration of the cornea in the main incision, the eye was left somewhat hypotonic, no intracameral medication was used, no air bubble was left in the anterior chamber, and a few drops of Vigamox® were dripped onto the eye with a subsequent occlusive dressing. The actual surgical time, from the initial corneal incision to completion with Vigamox eye drops, can be considered the same for both surgeries, not exceeding 15 min.

On postoperative day (POD) 2 of the OD, the patient presented with acute onset of severe vision loss acuity. Ophthalmologic examination showed BCVA of counting fingers at 50 cm in OD and 1.0 in OS. Biomicroscopy of the anterior segment revealed no changes, and fundoscopy was unremarkable, except for a slight macular paleness of OD, which motivated further imaging investigation.

## DISCUSSION

The patient had a history of vascular system abnormalities, which are believed to be the main risk factor for PAMM, regardless of any surgical scenario. In this case, chronic vasculopathies can be considered more severe and had a higher risk for worse outcomes because of the patient’s age, time of disease progression, and significant multiple target organ damage (heart, vessels, and kidneys).

Before cataract surgeries, the patient had echodoppler cardiogram, which showed no signs of thrombus or intracavitary masses, and *24-h Holter* monitoring that did not reveal arrhythmia. The patient was using anticoagulants because of a previous myocardial infarction. All these suggest that in this case, PAMM may have been favored by another factor during surgery rather than a thromboembolic phenomenon.

The surgery and anesthetic procedures were performed identically in both eyes. Thus, some factors may have contributed to the increase in IOP and/or increased risk of PAMM development perioperatively. Peribulbar anesthesia may have increased the IOP by mechanical compression of the intra-orbital and intraocular vessels^(15)^ and the weight positioned over the eye could also have increased the IOP by a similar mechanism^(15)^. Prolonged pressure against the globe increases the intraorbital pressure beyond the intraluminal values, resulting in the compression of retinal arteries and ciliary vascular system. The use of a high infusion pressure into the eye increased the IOP during surgery. Although usua lly controlled during surgery, even moderate infusion pressure could have generated low perfusion pressure in the retinal arteries of a susceptible eye patient. Moreover, diluted concentrations of lidocaine and ropivacaine used in peribulbar anesthesia may have provided vasoconstrictor effects^(16)^, whereas venous sedation could have generated arterial hypotension.

**Figure 1:**
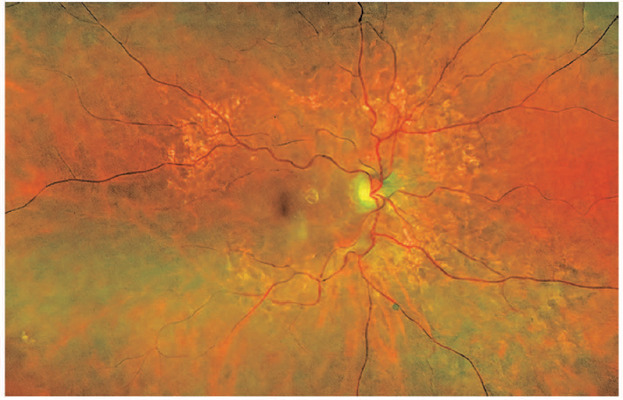
Color retinography of the right eye. Fundus photograph represents yellow small dots scattered throughout the posterior pole of the eye, concentrated around the papilary area, suggesting drusen. No other lesions can be noted at the retina, vessels, or optic nerve head.

**Figure 2:**
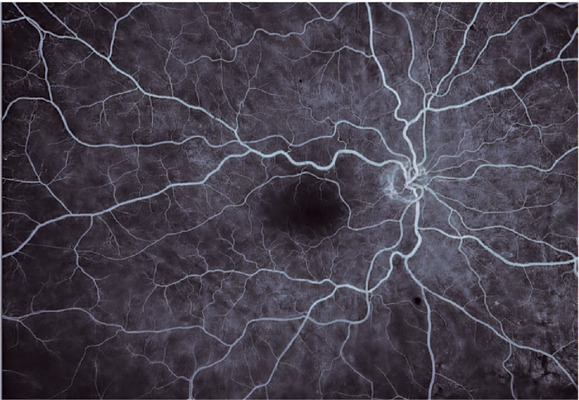
Right eye fluorescein angiogram showing the absence of leakage or ischemia in the macular area or vascular occlusions. The optic disk head shows normal fluorescence pattern with no lesions noted. Drusens appear as hyporeflective dots during the contrast phase.

**Figure 3:**
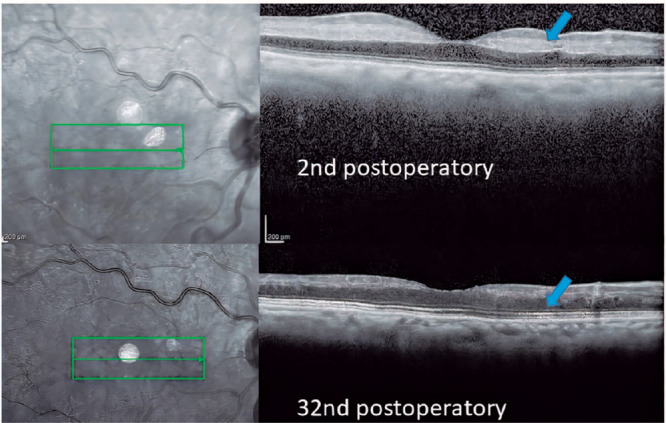
Right-eye spectral-domain optical coherence tomography (SD-OCT) on postoperative day 2 of OD phacoemulsification. SD-OCT shows paracentral placoid, hyperreflective bands at the inner nuclear layer (arrows) sparing the outer retina in the right eye, consistent with paracentral acute middle maculopathy (PAMM). Right-eye SD-OCT on postoperative day 32 of phacoemulsification reveals thinning/atrophy of the middle retinal layers in the distribution of previous PAMM lesions. Other retinal layers were apparently spared.

Fundoscopy (Figure 1) or fluorescein angiography (Figure 2) after the symptoms of vision loss in OD revealed no abnormalities, except for drusen scattered throughout the posterior pole. Nevertheless, SD-OCT findings were suggestive of PAMM, showing hyperreflective, band-like lesions in the middle retina, extending from the INL/outer plexiform layer junction to involve the full-thickness INL (Figure 3). Over time, these lesions resolve with INL atrophy. Those changes are highly suggestive of ischemic insult to the inner retina^(1)^. According to a recent publication^(14)^, PAMM should be the main diagnostic hypothesis in patients who present with severe vision loss and unremarkable ophthalmologic examination soon after undergoing non-complicated cataract surgery. Even after an extended follow-up period, the patient experienced persistent paracentral scotoma and low vision acuity without further improvement, which was most likely due to INL atrophy. PAMM is a clinical finding suggesting ischemic insults to the intermediate and deep capillary plexus layer of the retina and is probably far more common than we could diagnose before the SD-OCT era. Currently, no treatment has been available; therefore, management should be targeted toward controlling systemic risk factors and reducing possible vascular insults during ocular surgical procedures.

PAMM can cause severe eye disorders after successful cataract surgery. Risk factors intrinsic to the patient and the anesthetic and surgical procedure should be carefully individualized to, if possible, prevent or minimize and/or minimize PAMM-induced damage.
